# Development of Oncolytic Vectors Based on Human Adenovirus Type 6 for Cancer Treatment

**DOI:** 10.3390/v15010182

**Published:** 2023-01-07

**Authors:** Ivan D. Osipov, Valeriia A. Vasikhovskaia, Daria S. Zabelina, Sergei S. Kutseikin, Antonina A. Grazhdantseva, Galina V. Kochneva, Julia Davydova, Sergey V. Netesov, Margarita V. Romanenko

**Affiliations:** 1 Faculty of Natural Sciences, Novosibirsk State University, 630090 Novosibirsk, Russia; 2 State Research Center of Virology and Biotechnology Vector, 630559 Novosibirsk, Russia; 3 Surgery Department, University of Minnesota, Minneapolis, MN 55455, USA

**Keywords:** adenovirus type 6 (HAdV-C6; Ad6), oncolytic virus, virotherapy, adenoviral genome modification, cloning

## Abstract

Human Adenovirus type 6 (HAdV-C6) is a promising candidate for the development of oncolytic vectors as it has low seroprevalence and the intrinsic ability to evade tissue macrophages. However, its further development as a therapeutic agent is hampered by the lack of convenient cloning methods. We have developed a novel technology when a shuttle plasmid carrying the distal genome parts with modified E1A and E3 regions is recombined in vitro with the truncated HAdV-C6 genome. Using this approach, we have constructed a novel Ad6-hT-GM vector controlled by the hTERT promoter and expressing granulocyte-macrophage colony-stimulating factor (GM-CSF) instead of 6.7K and gp19K E3 proteins. We have demonstrated that control by the hTERT promoter may result in delayed viral replication, which nevertheless does not significantly change the cytotoxic ability of recombinant viruses. The insertion of the transgene by displacing the E3-6.7K/gp19K region does not drastically change the expression patterns of E3 genes; however, mild changes in expression from major late promoter were observed. Finally, we have demonstrated that the treatment of human breast cancer xenografts in murine models with Ad6-hT-GM significantly decreased the tumor volume and improved survival time compared to mock-treated mice.

## 1. Introduction

Oncolytic viral therapy is a promising strategy in cancer treatment. To date, many oncolytic viruses have reached the third stage of clinical trials with two drugs already approved for clinical use: Imlygic, a recombinant herpes simplex virus-1 approved by the US Food and Drug Administration for treating advanced melanoma; and Oncorine, an adenovirus-based vector for treating nasopharyngeal carcinoma in China [1,2]. Among all the viral families exploited as backbones for oncolytic vector development, adenoviruses (Ads) are one of the most well-studied and promising group.

The most well-known and studied oncolytic Ad backbone is adenovirus type 5 (HAdV-C5, designated Ad5 in this article). Many Ad5-based oncolytics undergo clinical evaluation; however, there are several obstacles which hamper the clinical translation of Ad5-based vectors. The most prominent obstacle is that Ad5 has the highest seroprevalence among human Ads, reaching up to 100% in some populations [3]. Additionally, after intravenous (i.v.) injection, Ad5 has been shown to be rapidly cleared by tissue macrophages which sufficiently restricts its tumor targeting and leads to severe hepatotoxicity in preclinical models [4]. It is well known that the most abundant capsid protein, hexon, is involved in multiple interactions with blood proteins and cellular receptors, which leads to rapid clearance. The approaches of hexon switching to that of other Ad types are developed to abolish these interactions. However, these modifications often lead to deficient virion maturation, increased toxicity, and difficulties with virus amplification and manufacturing [5,6,7]. An alternative way to address the obstacles of Ad5-based oncolytics is to develop new Ad backbones completely based on alternative Ad types with less seroprevalence and different biodistribution profiles, allowing better tumor targeting.

HadV-C6 (Ad6) belongs to the same species C of the *Adenoviridae* family as Ad5; however, its hexon protein structure strongly differs from Ad5. It is opsonized by immunoglobulins and other blood proteins in a different manner from Ad5 and allows to avoid rapid trapping by tissue macrophages [4,8]. Moreover, Ad6 is known to have lower seroprevalence relative to Ad5 [3] with no cross-reactivity between anti-Ad5 and anti-Ad6 neutralizing antibodies [9]. Considering all these properties, Ad6 is a highly prospective type for both locally and systemically delivered virotherapy.

Several studies have already demonstrated the promise of Ad6 towards various types of tumors, such as breast, ovarian, kidney, and liver [10,11,12]. We have recently shown the oncolytic potential of Ad6 against glioblastoma and its role to affect the cancer stem cells [13]. However, further genome modifications are needed to enhance Ad6 specificity and oncolytic efficacy. In this work, we first incorporated a tumor-specific human telomerase promoter, hTERT, to restrict the replication of virus to cancer cells [14]. Secondly, we inserted the GM-CSF transgene, prominently used as a therapeutic mono-agent and an enhancer for various oncolytic viruses, which magnifies the antitumor immune response [15,16]. To perform these modifications, we developed a novel strategy which is solely based on in vitro recombination and is not dependent on the specific *E. coli* strains. We then analyzed the influence of insertions on the recombinant virus replication in various cancer cell lines and estimated the expression of GM-CSF transgene. Finally, we assessed the oncolytic potential of new recombinant Ad6 vectors in vitro and in vivo.

## 2. Materials and Methods

### 2.1. Generation of Recombinant Ad6 Vectors

All recombination procedures and amplification steps were performed with the In-Fusion HD cloning kit and the Clone Amp HiFi PCR premix, respectively (Takara Bio, Kusatsu, Shiga, Japan). We first constructed the shuttle plasmid containing the left and right distal parts of Ad6 genomes with the sequences of interest (hTERT and GM-SCF). For that, we amplified the left part of the Ad6 genome (1–991 bp, includes left ITR, packaging signal, and partial E1A gene) using the primers Ad6-LITR-for and Ad6-LITR-rev (Table S1) and the right part of Ad6 genome (28,534–35,759 bp, from 5′-UTR of E3-6.7K to right ITR) using the primers Ad6RITR for and Ad6-RITR-rev (Table S1). Then, we recombined PCR-fragments with pBR322 digested by EcoRI and HindIII resulting in pShuttle-AdITRs. AsiSI sites were incorporated into primers Ad6-LITR-for and Ad6-RITR-rev so that they flanked the ITRs for the following release of a modified genome from the full-genome plasmid. After that, the native E1A promoter (360–559 bp) was substituted by hTERT promoter (from pAdV-TERT plasmid #26744, Addgene) using primers TERT-pShuttle-for and TERT-pShuttle-rev (Table S1) for the amplification of hTERT and pShuttle-TERT-for and pShuttle-TERT-rev (Table S1) for the amplification of pShuttle resulting in pShuttle-hTE1A-AdITRs. At the last step, a region containing two ORFs, E3-6.7K/gp19K (660 bp), was substituted with the GM-CSF gene obtained by PCR amplification from plasmid pCMV-GMCSF kindly provided by Dr. Kochneva (The State Research Center of Virology and Biotechnology “Vector”) with primers GMCSF-pShuttle-for and GMCSF-pShuttle-rev for the pShuttle and pShuttle-GMCSF-for and pShuttle-GMCSF-rev (Table S1) for the GMSCF resulting in pShuttle-hTE1A-AdITRs-GMCSF plasmid. Finally, pShuttle-hTE1A-AdITRs-GMCSF was amplified with primers pShuttle-ClaI-Ad6 and pShuttle-PacI-Ad6 and recombined with the Ad6 genome digested with ClaI and PacI. Modified Ad6 genome was digested from a full-genome plasmid with AsiSI and transfected to A549 cells to rescue the virus.

All viruses were propagated in A549 cells in Dulbecco’s modified Eagle medium, DMEM (Capricorn, Ebsdorfergrund, Germany) supplied with 5% fetal bovine serum, FBS (Biowest, Nuaillé, France), harvested and purified in CsCl-gradient centrifugation. Viral prep was dialyzed using Slide-A-Lyzer cassettes 10K MWCO (Thermo Fisher Scientific, Waltham, MA, USA) and then preparations were supplemented with sterile glycerol to a final concentration of 7%, aliquoted and stored at −80 °C. The viral copy number was determined as described earlier [17]. Shortly, viral sample was diluted 1:1 with the lysis buffer (1% SDS, 2 mM EDTA in PBS) and incubated at 56 °C in a water-bath for 10 min. Then optical density was measured at 260 nm and the titer was counted by the formula: VP/mL = 0.955 × 2 (dilution factor) × 10^12^.

### 2.2. Cells Lines

A549 (a human lung epithelial cell line) cell line was kindly supplied by The State Research Center of Virology and Biotechnology “Vector”. U-87 MG (a human glioblastoma astrocytoma cell line), U-251 MG (a human glioblastoma astrocytoma cell line) and HepG2 (a human hepatocellular carcinoma cell line) cell lines were kindly gifted by the SPF-vivarium of the Institute of Cytology and Genetics SB RAS (Novosibirsk, Russia). MCF-7 (a human breast carcinoma cell line) and MDA-MB-231 (a human triple-negative breast adenocarcinoma cell line) cell lines were kindly provided by Joint Center for genomic, proteomic and metabolomics studies ICBFM SB RAS (Novosibirsk, Russia). A549, HepG2, MDA-MB-231, U-87 MG, and U-251 MG cells were cultured in DMEM (Capricorn Scientific, Germany) and MCF-7 cells were cultured in 5% IMDM (Capricorn Scientific, Germany) supplemented with 5% FBS, 100 μg/mL streptomycin, 100 U/mL penicillin and 250 ng/mL Amphotericin B at 37 °C and 5% CO_2_.

### 2.3. Cell Viability Assays

The cytotoxic effect of the recombinant adenoviruses was studied on a panel of human tumor cells: A549, HepG2, MDA-MB-231, U-87 MG, U-251 MG, and MCF-7. To determine viral dose-dependent activity, cells were seeded on a 24-well plate at a density of 5 × 10^4^ cells/well. The next day, cells were infected with viruses at the multiplicities of infection (MOIs) of 0.01, 0.1, and 1 vp/cell for HepG2 and MDA-MB-231, and 0.1, 1, and 10 vp/cell for other cell lines. After an 8-day incubation, the medium was removed, and cells were fixed with 10% buffered formaldehyde PBS and stained with 1% crystal violet in 70% ethanol. The plates were washed, dried, and observed.

To test the time-dependent oncolytic activity, cells were seeded on a 96-well plate at a density of 10^4^ cells/well. The next day, cells were infected with the viruses at MOI of 100 vp/cell or with a mock medium for negative control and incubated at 37 °C and 5% CO_2_ for various time spans. Cell viability was estimated each 48 h by the CyQUANT™ XTT Cell Viability Assay (Thermo Fisher Scientific, USA) according to the manufacturer’s guidelines. The optical density OD450/655 was measured on an iMark Microplate Absorbance Reader (BioRad, Hercules, California, DC, USA). Optical density in infected wells was calculated as % in relation to a negative control.

### 2.4. In Vivo Study

Six weeks old female SCID mice were housed under specific pathogen-free (SPF) conditions. The MDA-MB-231 tumor models were generated by implantation of 3 × 10^6^ cells subcutaneously into SCID mouse flanks with Matrigel to support growth. In tumor treatment studies, mice were treated with Ad5, Ad6, Ad6-hT-GM (1 × 10^10^ vp/mouse, *n* = 6–7 per group) or vehicle control (PBS, *n* = 6 per group) via intratumoral (i.t.) injection on days 1, 3, 5. Tumor growth was measured in two dimensions, recording the greatest length and width using calipers. Tumor sizes were plotted as the average size for each group. Mice were euthanized after tumors reached a volume of 1800 mm^3^ or when tumors displayed ulceration. All experiments with animals were conducted in strict compliance with the principles of humanity in accordance with the European Community Council Directives (86/609/EEC) and were approved by the Animal Care and Use Committee of the Institute of Cytology and Genetics SB RAS.

### 2.5. Biological Activity of GM-CSF

Medium of A549 infected with Ad6-hT-GM at MOI = 100 vp/cell and non-infected A549 cells were collected 48 hpi (hours post-infection) and clarified by centrifugation for 10 min at 2800× *g*. It was filtered then through the Amicon Ultra 100 K filter unit (Millipore Burlington, MA, USA) to remove the virus. Two-fold dilutions of the culture medium in the range of 1:4 to 1:1024 were prepared for the experiment in RPMI medium (Invitrogen Waltham, MA, USA) supplemented with 10% fetal calf serum (HyClone, Logan, UT, USA).

### 2.6. ELISA Assay

To determine active time-dependent GM-CSF secretion, A549 cells were seeded in wells of 6-well plate and 24 h later infected with Ad6-hT-GM at MOI of 100 vp/cell. The medium was changed, and the cells were incubated in fresh medium for 1 h at different time points and then collected.

To test the cumulative secretion of GM-CSF, MDA-MB-231, U-87 MG, and A549 cells were seeded in wells of 6-well plate and infected with Ad6-hT-GM 24 h late at MOI of 100 vp/cell. The medium was collected at various time points.

Collected media samples for both experiments were centrifuged to pellet cells and 100 μL of supernatant were probed for analysis. The GM-CSF content was measured using an ELISA kit for Colony Stimulating Factor 2 (Cloud-Clone Corp, Houston, TX, USA) according to the manufacturer’s guidelines.

### 2.7. RT-qPCR for Viral mRNA

A549 cells were seeded in 6-well plate and infected with Ad6, Ad6-hT, or Ad6-hT-GM 24 h late at MOI of 100 vp/cell. The cells and medium were collected by centrifuging. The lysis buffer from Ribo-prep kit (Amplisens, Moscow, Russia) was added to plate wells and reunited with pellet cells. Finally, total nucleic acid extraction was conducted according to the manufacturer’s recommendation.

Samples were treated with DNase I to get rid of genomic DNA before proceeding to analysis (Thermo Fisher Scientific, Waltham, MA, USA). Reverse transcription was performed with oligo-dT primer using an MMLV reverse transcription kit (Evrogen, Moscow, Russia). Quantitative PCR was performed on the CFX96 Touch Real-Time PCR Detection System. Specific primers and the probe (Table S1, primers #15–20) were designed to amplify E3 genes 6.7K, GM-CSF and ADP from the E3 promoter or major late promoter (MLP).

All reaction mixes were prepared using 5X qPCRmix-HS (Evrogen, Russia) with 0.4 μM of primers and 0.25 μM of probe in a final volume of 15 μL. The following qPCR thermoprofiles were used: initial activation at 95 °C for 5 min, followed by 45 cycles of 95 °C for 30 s, 55 °C for 10 s and 70 °C for 40 s with a signal acquisition in the Cy5 channel at the end of the annealing/extension step. Each reaction was run in triplicate. The Cq values were estimated by analyzing the data using the threshold and baseline cycle option of the CFX Manager Software v3.1. Data were normalized to the GAPDH expression level (Table S1, primers #21–23).

### 2.8. Quantification of Ad by qPCR

To test adenoviral replication dynamics samples were collected and total nucleic acid was extracted as described for RT-qPCR experiments.

To determine DNA viral copy number, lung, spleen, and liver tissues from sacrificed animals (60–120 mg) were placed into homogenization microtubes with the 200 µL PBS and homogenized with MagNA Lyser (Roche Diagnostics, Basel, Switzerland). Total DNA was extracted using Ribo-prep DNA extraction kit (Amplisens, Moscow, Russia) according to the manufacturer’s guidelines. Precipitated DNA was further dissolved in MQ-quality water.

The total amount of DNA in samples was assessed on QUBIT fluorometer (Thermo Fisher Scientific, USA). Quantitative PCR was performed on the CFX96 Touch Real-Time PCR Detection System. All the reaction mixes were prepared using BioMaster HS-qPCR(2x) Kit (Biolabmix, Novosibirsk, Russia) with 0.4 μM of primers and 0.25 μM of a probe in a final volume of 15 μL. Specific primers and the probe (Table S1, primers #24–26) were designed to amplify E4 region.

The following qPCR thermoprofiles were used: initial activation at 95 °C for 5 min, followed by 45 cycles of 95 °C for 25 s and 58 °C for 20 s with a signal acquisition in the Cy5 channel at the end of the annealing/extension step. For each experiment, the approximating function based on standardized dilutions of the viral DNA (10^2^–10^8^ copies per reaction) was plotted and used for the calculation of the target DNA template in the reaction. Each reaction was run in triplicate. The Cq values were estimated by data analysis using the threshold and baseline cycle option of the CFX Manager Software v3.1.

### 2.9. Statistical Analyses

We used R programming environment for statistical analyses [18]. Statistical significance for difference in viral copy numbers, gene expression, the data from cell viability experiment and viral burden was assessed using ANOVA followed by Tukey HSD test for pairwise comparisons between groups. Survival data were analyzed by log-rank test. For the tumor size and viral burden, statistical significance was calculated using Mann–Whitney U test with Bonferroni correction. *p*-values of < 0.05 were considered statistically significant.

## 3. Results

### 3.1. Construction of the Recombinant Ad6-Based Vectors

We made two modifications of the Ad6 genome: the replacement of the native adenovirus E1A gene promoter with the hTERT one, and the insertion of the human GM-CSF gene into the E3 region. GM-CSF transgene was inserted by replacing ORFs–E3-6.7K and E3-gp19K, so that adenovirus death protein (ADP) and E3B region (RIDα/β, and E3-14.7k genes), which deletion leads to poor viral spread and fast virus clearance, are left intact [19,20].

**Figure 1:**
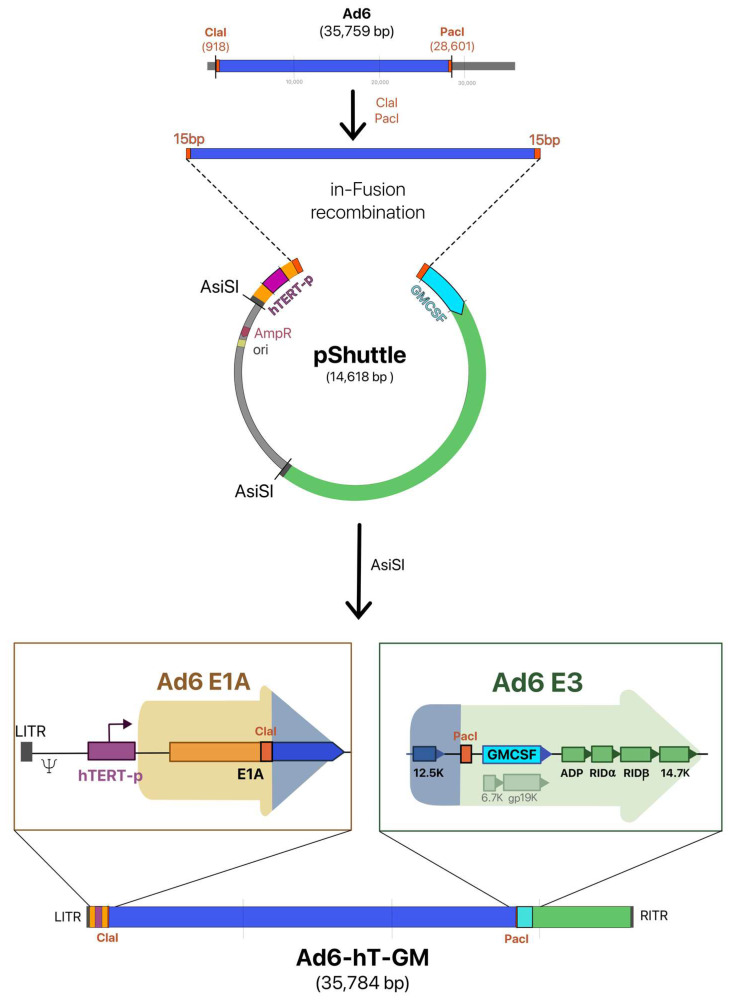
Construction of Ad6-hT-GM. pShuttle is based on pBR322 and contains the left part of the Ad6 genome from LITR to 1773 bp (shown in yellow) with hTERT promoter (shown in purple) replacing the native E1A promoter and the right part, from 27,576 bp to RITR (shown in green) with the GM-CSF transgene inserted instead of E3-6.7K and E3-gp19K genes (shown in cyan). pShuttle was linearized by amplification with primers containing 15 nucleotide homology arms (shown in orange). The Ad6 genome was digested with ClaI and PacI, and recombination between truncated genome and linearized pShuttle was performed by the In-Fusion kit. Ad6-hT-GM recombinant virus genome was then retrieved from the plasmid with the AsiSI. Expanded regions indicate modified Ad6-hT-GM genome regions.

The most common method for generating recombinant Ads remains homologous recombination in vivo using *E. coli* strain BJ5183 [21]. However, many new convenient approaches have been developed to facilitate both vectorization and modifications of Ad genomes based on homologous recombination in vitro. Thus, the In-Fusion kit, allowing for rapid cloning of DNA sequences, has already been used for the vectorization of Ad6 with all the subsequent modifications performed by standard restriction enzyme cloning [22]. As the Ad loci that need to be modified are located at the distal parts of the genome, and most of the proximal genome DNA (about 26 kb) remains intact, we have not performed the primary genome vectorization in our approach. Instead, at the first step, the shuttle plasmid (pShuttle-hTE1A-AdITRs-GMCSF), which contains the distal parts of the genome with modified E1 and E3 regions, was constructed. Then, the recombination of pShuttle-hTE1A-AdITRs-GMCSF with the truncated Ad6 genome was performed (Figure 1).

pShuttle-hTE1A-AdITRs-GMCSF contains the left distal part of the Ad6 genome (1–1773 bp) with the hTERT promoter inserted upstream of the E1A gene and the right distal part of the genome (27,576–35,759 bp) with the GM-CSF transgene inserted instead of E3-6.7K and E3-gp19K genes. The pShuttle-hTE1A-AdITRs-GMCSF was amplified using primers containing 15 base sequences homologous to the distal parts of the Ad6 genome digested with ClaI and PacI restriction enzymes with subsequent homologous recombination by In-Fusion recombinase. This final step is visualized in Figure 1. Ad6-hT-GM recombinant virus genome was then excised with the AsiSI and rescued in A549 cells.

Ad6-hT was constructed by the same approach with no GM-CSF insertion.

### 3.2. The Influence of hTERT-Promoter Insertion on Adenoviral Replication In Vitro

**Figure 2:**
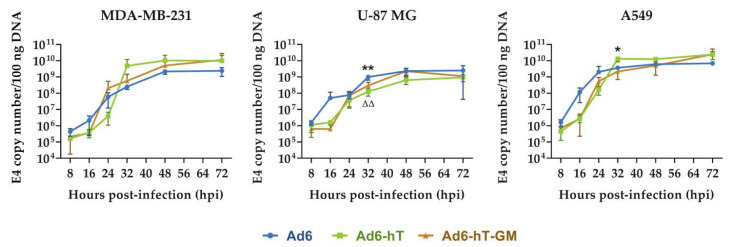
The influence of genome modifications on the rate of adenoviral replication in various cell lines. MDA-MB-231, U-87 MG, and A549 were infected with Ad6, Ad6-hT, and Ad6-hT-GM with MOI = 100 vp/cell, and DNA copy numbers were assessed at various time points by qPCR with primers for adenoviral E4 region. Data are presented as mean ± SD (*n* = 3). The differences between viral copy numbers were analyzed by ANOVA followed by Tukey’s HSD post-hoc test for pairwise comparisons. * *p* < 0.05 and ** *p* < 0.01 denote significant differences between Ad6-hT and Ad6. ΔΔ *p* < 0.01 denotes significant difference between Ad6-hT-GM and Ad6.

We analyzed the impact inserted modifications have on adenoviral replication. Three cell lines (MDA-MB-231, U-87 MG, and A549) were infected with Ad6, Ad6-hT, and Ad6-hT-GM with MOI = 100 vp/cell, and DNA copy number was assessed at various time points (Figure 2). A mild delay of recombinant viruses replication compared to the wild-type Ad6 was observed at 16 h post-infection (hpi) in U-87 MG and A549 cells; however, the differences in DNA copy numbers were not significant. The only significant difference which was observed between recombinant and wild-type viruses was at 32 hpi in U-87 MG cells (*p* = 0.008 for both Ad6-hT-GM and Ad6-hT compared to Ad6); however, no difference was detected at later time points. Surprisingly, Ad6-hT vector had significantly higher DNA copy number compared to wild-type Ad6 at 32 hpi in A549 cells (*p* = 0.015); this difference also disappeared at later time points. The initial viral copy numbers were significantly lower for MDA-MB-231 for all analyzed viruses compared to U-87 MG and A549 (*p* = 0.0012 and 0.0091, respectively). However, the plateau level (calculated as a mean value at 72 h for all three viruses) for A549 was the highest (9 × 10^9^ DNA copy numbers/100 ng DNA), intermediate for MDA-MB-231 (3.5 × 10^9^), and the lowest for U-87 MG (9.6 × 10^8^). For all analyzed vectors, the number of viral genome copies stopped increasing between 32 and 48 h, which corresponds to the complete Ad6 life cycle length [23].

### 3.3. GM-CSF Expression in Ad6-hT-GM-Infected Cells

**Figure 3:**
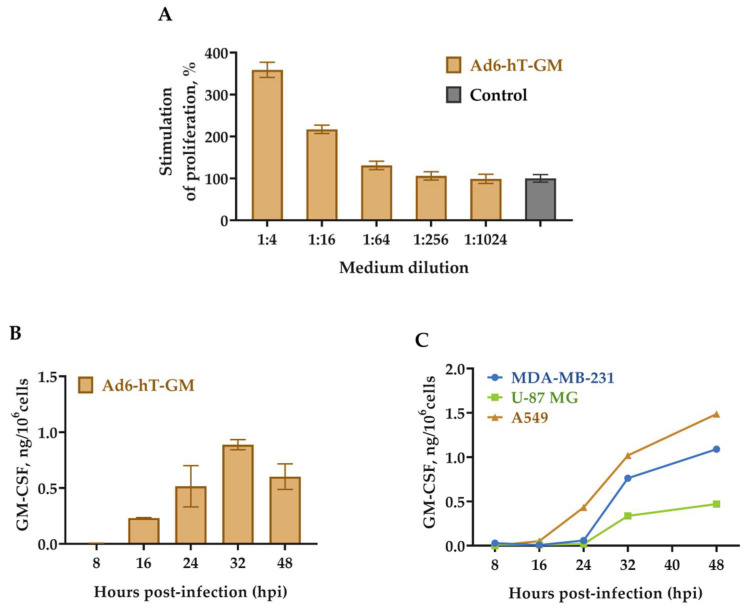
GM-CSF production by Ad6-hT-GM-infected cells. (**A**) Stimulation of TF-1 cells proliferation by GM-CSF. A549 cells were infected with Ad6-hT-GM at MOI = 100 vp/cell, supernatant was collected after 48 h. Supernatant collected from mock-infected cells was used as a control. Stimulation of TF-1 cells proliferation was determined by XTT assay following a 72 h incubation. Data from the mock-infected group was normalized to 100%. Data are presented as mean ± SD (*n* = 3). (**B**) Dynamic of GM-CSF expression in A549 cells. Cells were infected with Ad6-hT-GM (MOI = 100 vp/cell); at various time points, infected cells were incubated with fresh medium for 1 h before collection and measured for GM-CSF concentration by ELISA. Data are presented as mean ± SD (n = 3). (**C**) Dynamic of GM-CSF production in various Ad6-hT-GM-infected cell lines. A549, MDA-MB-231, and U-87 MG cells were infected with MOI = 100 vp/cell; medium was collected, and GM-CSF concentration was measured by ELISA.

First, we assessed the biological activity of the GM-CSF secreted from Ad6-hT-GM using the TF-1 cells (Figure 3A). TF-1 is a human erythroleukemia cytokine-dependent cell line that proliferates only in the presence of human GM-CSF or IL-3. The filtered medium of the A549 cells infected with the Ad6-hT-GM was serially diluted and added to the TF-1 cells, whose proliferative response was evaluated by the XTT test. TF-1 cell viability increased in a dose-dependent manner, confirming the presence of biologically active GM-CSF in the medium of Ad6-hT-GM-infected A549 cells.

Then, we determined the kinetics of GM-CSF expression in the medium of Ad-hT-GM-infected A549 cells (MOI = 100 vp/cell, Figure 3B). Infected cells were incubated with fresh medium for 1 h before collection, and GM-CSF concentration was measured by ELISA. GM-CSF secretion started between 8 and 16 hpi and decreased after 32 h, which corresponds to the beginning of the cell lysis.

Interestingly, when the GM-CSF accumulation was measured in various cell lines (MDA-MB-231, U-87 MG, and A549), A549 appeared to be the most productive, MDA-MB-231 an intermediate, and U-87 GM the least productive cell line, which corresponds to the viral specific growth rates in these cell lines (Figure 3C).

### 3.4. The Influence of Ad6 Genome Modification on the Expression of E3-Coded Genes

**Figure 4:**
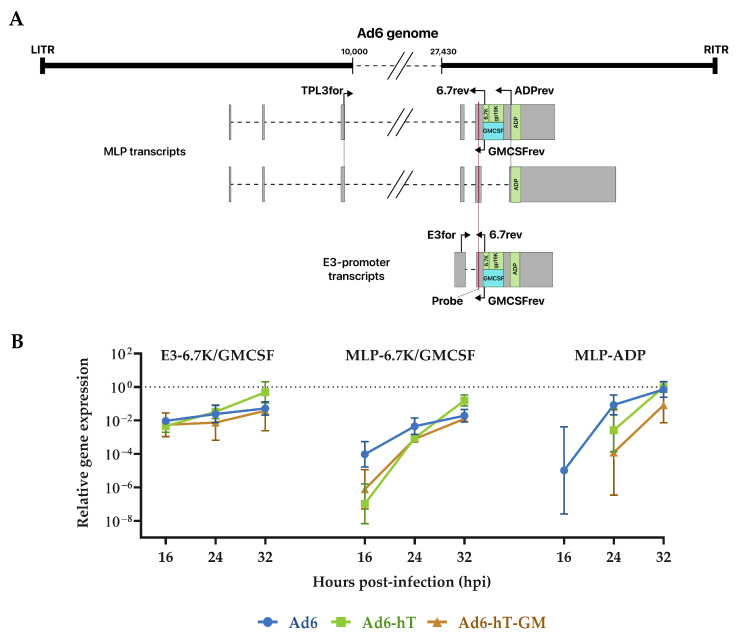
Analysis of E3-coded genes expression. (**A**) Scheme of E3 mRNA transcripts and primer design. (**B**) The relative expression of E3-6.7K, ADP genes and GM-CSF transgene. A549 cells were infected with Ad6, Ad6-hT, and Ad6-hT-GM with MOI = 100 vp/cell, total RNA was collected, and mRNA transcripts copy number was assessed by RT-PCR and normalized to the GAPDH expression level. E3-6.7K/GMCSF–RT-PCR results with primers E3for and 6.7rev/GMCSFrev for Ad6, Ad-hT/Ad6-hT-GM respectively, MLP-6.7K/GMCSF–TPL3for and 6.7rev/GMCSFrev, MLP-ADP–TPL3for and ADPrev, the probe was the same for all the settings. Data are presented as mean ± SD (*n* = 3). The differences between the relative expression levels were analyzed by ANOVA followed by Tukey’s HSD post-hoc test for pairwise comparisons.

It was previously shown that the expression patterns of E3-coded genes and transgenes are dependent on the locus of transgene insertion [24,25,26]. We studied the expression of GM-CSF in A549 cells infected with recombinant Ad6-based vectors compared to wild-type Ad6 at various time points. For that, we analyzed mRNA transcripts containing 6.7K/gp19K ORFs in wild-type Ad6 and Ad6-hT and mRNA transcripts containing GM-CSF in Ad6-hT-GM by RT-PCR. As transcription of E3 genes initiates from both E3 and major late promoter (MLP), we used forward primer specific to the common 5′UTR for detection of E3 promoter-initiated transcripts and primer specific to the third part of tripartite leader (TPL) to detect MLP transcripts (Figure 4A). Besides, we analyzed the expression of ADP by assessing MLP transcripts containing ADP ORF. The Ad5 transcription and splicing map was used as a reference [27].

As expected, the expression of GM-CSF for Ad6-hT-GM and 6.7K/gp19K for Ad6-hT and wild-type Ad6, respectively, was found mostly from the E3 promoter and to a lesser extent from MLP (Figure 4B). Under the control of E3 promoter, the expression of native E3 genes and transgene had no difference. The expression of 6.7K/gp19K from MLP was higher than GM-CSF at 16 hpi; however, that difference was not significant. ADP expression in wild-type Ad6 started earlier compared to recombinant viruses, which corresponds to the replication delay in A549 (see Figure 2).

### 3.5. Oncolytic Efficacy of Ad6-hT-GM In Vitro

**Figure 5:**
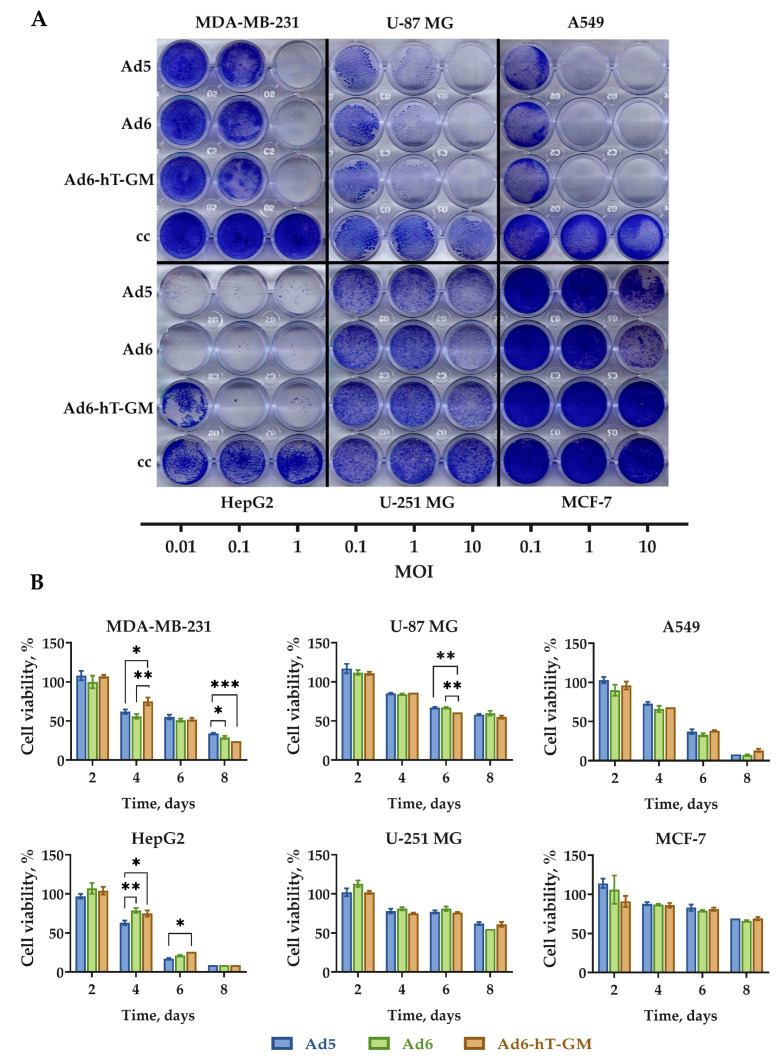
Comparison of the in vitro oncolytic effect of Ad5, Ad6 and Ad6-hT-GM in various cell lines. (**A**) Cells were infected at 0.1–10 vp/cell with the indicated viruses, 8 days post-infection (dpi) cell viability was assessed by crystal violet assay. (**B**) Cells were infected at 100 vp/cell with the indicated viruses, and cell viability was measured 2, 4, 6, and 8 dpi by XTT assay. Data are presented as mean ± SD (*n* = 3). The differences between cell viability levels were analyzed by ANOVA followed by Tukey’s HSD post-hoc test for pairwise comparisons. * *p* < 0.05, ** *p* < 0.01, *** *p* < 0.001.

To evaluate the cytotoxic effect of Ad6-hT-GM compared to the wild-type Ad5 and Ad6, a panel of human cancer cell lines of different origins were used: A549 (lung cancer), MDA-MB-231 and MCF-7 (breast cancer), U-87 MG and U-251 MG (glioblastoma), and HepG2 (hepatocarcinoma). Cell viability was determined by the crystal violet assay on the 8th day post-infection, dpi (Figure 5A). HepG2 showed the highest sensitivity to all three tested viruses among all cell lines while A549 and MDA-MB-231 cells were less sensitive. However, the wild-type Ads cytotoxicity overcame Ad6-hT-GM on the HepG2 and MCF-7 cell lines, suggesting slight Ad6-hT-GM lytic ability attenuation level due to the hTERT/GM-CSF insertions.

To further test the dynamics of viral cytotoxicity, the same cell lines were infected at MOI of 100 vp/cell, and cell viability assay by XTT assay was measured every 48 h. As shown in Figure 5B, starting on day 4, all viruses demonstrate a prominent cytotoxic effect in all tested cell lines. In accordance with the crystal violet assay, the MCF-7 and U-251 MG cells showed almost no further cytotoxicity after day 4, thus being the least sensitive cell lines for Ad infection. HepG2, A549, and MDA-MB-231, on the contrary, showed the highest sensitivity towards Ad vectors. On days 4 and 6, the oncolytic activity of Ad6-hT-GM on the HepG2 cell line was significantly lower compared to the wild-type Ad5, but it equaled by day 8. For the U-87 MG cells, on the contrary, Ad6-hT-GM performed significantly better on day 6 than the wild-type Ads, but the cytotoxic effect became equal by day 8. In MDA-MB-231 cells Ad6-hT-GM, demonstrated its superior cytotoxic ability compared to wild-type Ad5 at the final time point.

### 3.6. The Influence of Genome Modification on Ad-hT-GM Lytic Ability In Vivo

**Figure 6:**
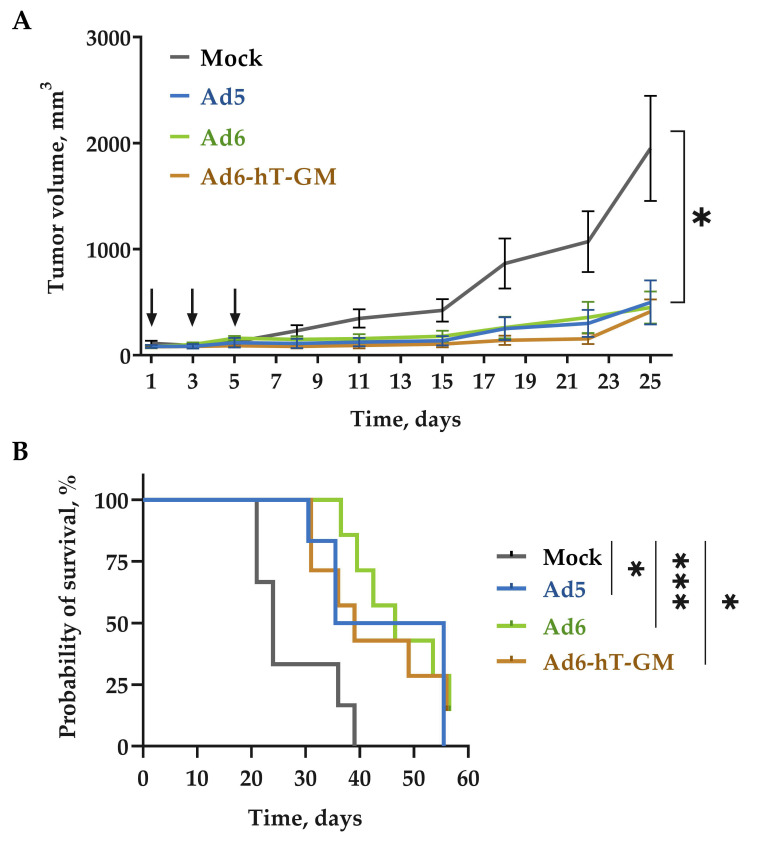
Oncolytic potency of adenoviruses on MDA-MB-231 bearing SCID mice. (**A**) Mean tumor volumes. SCID mice (*n* = 6–7) with preestablished MDA-MB-231 tumors (approximately 100 mm^3^) were intratumorally injected with 10^10^ vp of indicated viruses at day 1, 3, and 5 (indicated by arrows). Mann-Withney test was performed between control and the treated groups. The values are presented as mean ± SEM. (**B**) Survival data were depicted as Kaplan-Meier survival curves. Comparison between treated groups and the mock group was conducted by a log-rank test. * *p* < 0.05, *** *p* < 0.001.

To evaluate the oncolytic potential of Ad6-hT-GM and the possible negative influence of tumor-specific promoter and transgene insertions on the lytic ability of recombinant vector in vivo, SCID mice were subcutaneously (s.c.) transplanted with MDA-MB-231 cells. When tumors reached the volume of 75 ± 15 mm^3^, mice were divided into four groups, receiving three intratumoral (i.t.) injections of Ad6-hT-GM or wild-type viruses, Ad5 and Ad6 (1 × 10^10^ vp/mouse), or control (PBS), with three-day time intervals (Figure 6A).

Ad6-hT-GM outperformed both Ad6 and Ad5 wild-type vectors on day 18; however, this difference was not statistically significant. The same trend was observed on day 22. At the end of the experiment, on day 25, the average tumor size of Ad6-hT-GM-treated animals was the minimal among all the virus-treated groups (411 mm^3^ for Ad6-hT-GM versus 449 and 497 for Ad6 and Ad5, respectively). At the same time, the average tumor size of mock-treated mice on day 25 was 1950 mm^3^, which is almost 5 times more than the size of Ad6-hT-GM-treated tumors. Thus, recombinant Ad6-hT-GM vector demonstrated high oncolytic efficacy decreasing the tumor size significantly compared to the PBS-treated control.

**Figure 7:**
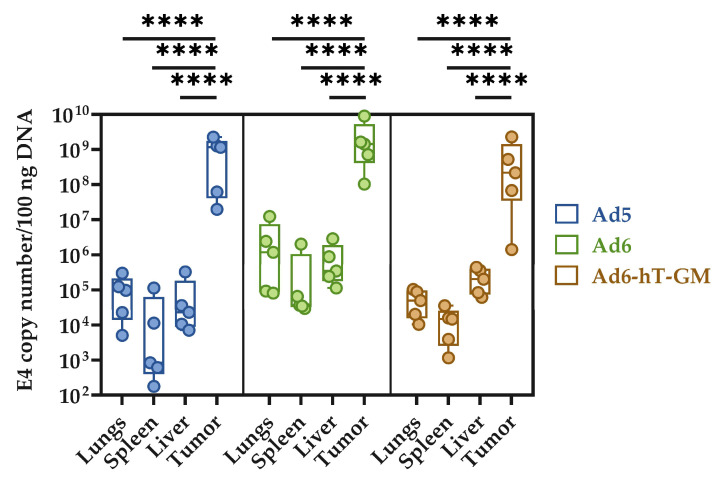
Adenoviral genomic DNA biodistribution in mice organs and tumors. E4 gene copy number per 100 ng of total DNA within the lung, spleen, liver, and tumor was evaluated using qPCR. The tumors demonstrated significantly higher DNA copy numbers compared to the organs (**** *p* < 0.0001). Data are presented as the Dot-boxplots, with *n* = 5 animals per group. The differences between cell viability levels were analyzed by ANOVA followed by Tukey’s HSD post-hoc test for pairwise comparisons.

After sacrificing the mice, tumor, liver, lungs, and spleen tissue samples were collected to evaluate the virus copy numbers (Figure 7). Although mice are not permissive to human Ad replication, the uptake of viruses by reticuloendothelial cells and the rate of cell transduction can be assessed. Viral DNA was detected in all analyzed organs indicating the virus spread upon i.t. injections. Importantly, the viral DNA copy numbers recovered from tumors were significantly higher than in any examined organ.

We also tested the survival of treated mice and found that treatment with Ad6-hT-GM significantly prolonged survival compared to mock-treated mice (Figure 6B). Although the median survival for Ad6-hT-GM was less than for wild-type viruses (39 days for Ad6-hT-GM versus 46 days for both Ad5 and Ad6), the difference was not significant. These studies suggest that performed modifications of Ad6 genome using our approach did not attenuate the oncolytic potential of Ad6-hT-GM.

## 4. Discussion

Ad6 has been shown to have oncolytic potential similar to Ad5 and, in some cases, even outperform other tested Ad types. Thus, it has been demonstrated that single treatment of tumors in Syrian hamsters with Ad6 effectively reduced kidney tumor xerographs growth in contrast to Ad5 and Ad11 [10,11,13]. Moreover, Ad6 is known to have lower seroprevalence compared to Ad5, which may result in a less intense neutralization by pre-existing antibodies upon systemic administration [3,28]. However, Ad6 is still considering to be the “alternative” type for oncolytic Ad vector development and is clearly underrepresented in the field of oncolytic virotherapy. There are only three recombinant Ad6-based oncolytic vectors reported so far: Ad6miR [22], Ad6d24.P19 [29], and Ad657 [30]. None of them are designed to control virus replication by the tumor specific promoter nor express the immunomodulatory transgene. As we recently reported, insertions of therapeutic transgenes and targeting cancer cells using tumor specific promoter have been rarely applied for Ad6-based vectors, mostly due to the lack of available cloning techniques to generate Ad6-based replication competent vectors [28].

In this work, we have applied a novel “one-step in vitro recombination” approach to perform genetic modifications of the Ad6 genome. First, we have inserted the hTERT promoter to control the E1A gene to improve vector specificity, which is crucial to restrict Ad replication to tumor cells. To enhance the antitumor efficacy, we inserted the immunotherapeutic gene, GM-CSF, responsible for the antigen-presenting cell activation. GM-CSF has been previously used in two of the most successful oncolytic agents—T-VEC and Pexa-Vec, based on the Herpes simplex virus and Vaccinia virus, respectively [31,32].

Since the packaging capacity of the adenoviral genome is limited to 105% [33], the deletion of non-essential viral genes is needed to insert exogenous DNA sequences. The E3 transcription unit of Ad species C codes for 12.5K, 6.7K, gp19K, adenoviral death protein (ADP), RID α and β, and 14.7K proteins (see Figure 1), which are considered non-essential for viral replication, and thus can be deleted to make space for therapeutic transgenes. Many oncolytic Ad5-based vectors were developed on the ΔE3 platform, which implies the deletion of the nearly entire E3 region to insert transgenes under either endogenous or exogenous promoters [34,35,36]. In some cases, it is more beneficial to delete ADP because it is a membrane protein that can negatively affect membrane-located transgenic proteins such as NIS [37]. However, ADP is more often reinserted to E3 deleted region as it leads to increased cell lysis and viral spread [19,38,39]. Despite the broad use of ΔE3 (with or without ADP) vectors, they were shown to be rapidly cleared by the immune system due to the immunosuppressive function of deleted E3 genes. Thus, Ad5 E3B genes (RIDα, RIDß, and 14.7K) are responsible for removing TRAIL or Fas receptors from the cell surface to protect the infected cell from apoptosis by lysis TNF and Fas [40,41]. E3-gp19K inhibits the transport of MHC I from the endoplasmic reticulum, preventing viral antigen presentation and host cell killing by CTL [42,43]. The E3-6.7K, together with E3B proteins, participates in degrading the TRAIL [41]. It was shown that deletion of both E3-6.7K and E3-gp19K led to more rapid elimination in immunocompetent Syrian hamsters compared to wild-type Ad5 [44]. Nevertheless, the E3B-deleted adenovirus has been found to be eliminated faster than the E3-gp19K-deleted Ad5 in the immunocompetent mouse model [20]. Considering all these previous observations, the E3-6.7K/gp19K region was deleted to provide the necessary space for transgene insertion in the current study. A similar approach has been used to construct Ad5-based vectors LOAd703 and ONCOS-102 [45,46].

Strategies of alternative Ad types genomes modification consist of two steps: genome vectorization, and full-genome plasmid modification. Both steps are usually performed by in vivo recombination in specific *E. coli* strains (mostly BJ5183), which codes for recombinase enzymes making full-genome plasmid unstable and difficult to handle. Dr. Ehrhardt’s group has suggested a new Ad vectorization method that simultaneously generates full-genome backbone plasmids for various Ad types [47]. However, this strategy still depends on unstable in vivo recombination methods in *E. coli*. Another strategy is based on seamless in vitro recombination systems when the parts of the Ad genome are subcloned, modified, amplified, and then enzymatically reassembled into the modified Ad genome [48,49]. This approach implies the construction of at least 7–9 plasmids/cosmids with overlapping junction regions for recombination which is error prone (e.g., for Gibson assembly, mutations observed in 10 to 20% of the constructs) [48].

Our new cloning strategy is based on the approach when instead of the enzymatic reassembly of the Ad genome, we can use two unique restriction sites, ClaI and PacI, which are conveniently located in E1A and E3 regions, respectively (see Figure 1). That allowed us to subclone the distal genome parts to the pShuttle and the regions of interest with the following recombination with the linearized pShuttle using the In-Fusion kit (of note, despite of manufacturer’s recommendations to use the kit for DNA fragment up to 10 kb, we proved it could be useful for the fragments up to 30 kb). Thus, this approach significantly minimizes the number of amplified fragments, leading to less mutation probability, and avoids laborious multi-step handling of the whole-genome plasmids after primary vectorization. Moreover, it is not dependent on specific *E. coli* strains. Our approach can be applied to other Ad types; if they do not have the appropriate restriction sites, the genome can be digested by enzymes such as EnGen^®^ Lba Cas12a.

Next, we analyzed the transgene expression with the recombinant Ad6-hT-GM vector in A549 cells. First, we showed that GM-CSF expressed by Ad6-hT-GM-infected A549 cells is biologically active. Second, we analyzed the dynamic of GM-CSF protein secretion by infected cells to the medium and showed that it peaked between 32 and 48 hpi, which is consistent with data previously obtained by Hawkins et al. for Ad5 with E3-6.7K/gp19K replaced with mTNF [24].

We have also analyzed the influence of Ad6 genome modification on the expression of E3-coded genes by RT-PCR. As GM-CSF transgene exactly replaces two open reading frames (ORFs), E3-6.7K and E3-gp19K, we compared the level of transcripts containing E3-6.7K and GM-CSF ORFs from both MLP and E3 promoter. For primer design, we used the Ad5 transcription and splicing map created by Donovan-Banfield et al. (see Figure 4A) [27]. Similar to Ad5, GM-CSF and 6.7K/gp19K in Ad6-based vectors were shown to be mostly expressed from E3 promoter and, to a lesser extent, from MLP. There was no significant difference in native E3-6.7K/gp19K and transgene expression while ADP expression of recombinant viruses was delayed compared to wild-type Ad6. It suggests minor influence of performed modifications to the E3-coded genes expression.

We analyzed the replication rates of the modified Ad6 vectors, Ad6-hT and Ad6-hT-GM, compared to the wild-type Ad6 in three different cell lines. There was no significant difference in the replication of recombinant viruses compared to the wild-type Ad6 in all tested cell lines except for one time point (32 hpi) in U-87 MG where the replication rate of wild-type Ad6 outperformed that of recombinant vectors. In addition, in A549 and U-87 MG cells, replication of hTERT-controlled recombinants tend to delay compared to wild-type Ad6 at 16 hpi. In the case of U-87 MG, it may be explained by the different expression of telomerase in these cell lines. While telomerase inhibition led to a decrease in the U-87 MG tumor size in vivo [50], the expression of active telomerase in U-87 MG in vitro was not detected [51] in contrast to MDA-MB-231 and A549, which were shown to express active telomerase [52,53]. In MDA-MB-231, the initial viral copy number was significantly lower for all the analyzed viruses compared to U-87 MG and A549, which is not surprising as MDA-MB-231 has been shown to have low CAR expression (only 5% of cell population express CAR) [54].

The observed replication rates correlate with the data showing the cytotoxic effect of Ad6-hT-GM. Thus, the U-87 MG plateau level for all the viruses being significantly lower compared to MDA-MB-231 and A549 correspond to the low lytic ability against U-87 MG cells. The overall cytotoxic activity of Ad6-hT-GM was similar to wild-type Ad6 as well as Ad5. However, in HepG2 and MCF-7 cell lines Ad6-hT-GM demonstrated a small degree of cytotoxic ability attenuation (mostly insignificant), which by the end of the observation period (day 8) ceased to be detected.

In many cancers, the antigen presentation machinery is dysregulated and even dysfunctional, allowing cancer cells to evade the immune response. Thus, the direct lytic ability of the oncolytic virus becomes crucial for effective therapy [55]. Therefore, we analyzed the influence of genome modification on the Ad6 direct lytic ability in vivo. For that, we used immunodeficient mice bearing human MDA-MB-231 breast cancer xenografts. We showed that the Ad6-hT-GM direct antitumor effect resembled that with wild-type Ad6 and Ad5. Ad6-hT-GM significantly decreased tumor growth compared to the PBS-treated group and even slightly outperformed the wild-type Ad6 and Ad5 control vectors on days 18 and 22 post-infection. In addition, Ad6-hT-GM significantly prolonged the survival of tumor-bearing mice compared to mock-treated animals. Importantly, immunodeficient murine model used in the current study are not suitable to demonstrate the full immunostimulatory potential of GM-CSF transgene expression in vivo. Hence, a further investigation of Ad6-hT-GM therapeutic efficacy in immunocompetent models is necessary.

The biodistribution analysis of Ad DNA in mice sacrificed 31–56 dpi showed the presence of viral DNA in the liver, spleen, and lungs. Of note, the virus uptake in normal organs was significantly lower compared to that in tumors. Besides, only small DNA fragments (68 bp) are detected by PCR, which may not represent the infectious viral particles. These data are consistent with the earlier studies demonstrating that i.t. injected Ad5-based vector can be detected in organs of immunodeficient mice with human xenografts with the level significantly lower than in tumors [56]. The shedding of the virus from the tumor site to distant organs can be speculated to occur due to extensive tumor necrosis followed by blood vessel damage and viral release to the bloodstream. It should be admitted that the distribution of human replication-competent Ads in murine models are restricted mostly to the uptake of viral particles by tissue macrophages, and cannot estimate the possible replication of the virus in mice organs.

Thus, the performed modifications of the Ad6 genome using our new cloning technology have had no negative effect on the oncolytic potential of Ad6-hT-GM recombinant virus. Our studies demonstrated that the insertion of both hTERT tumor specific promoter and GM-CSF transgene have not attenuated the performance of Ad6-hT-GM in vitro and in vivo models: its oncolytic efficacy was comparable with that of Ad6 and Ad5 wild-type control vectors.

## 5. Conclusions

In summary, this study offers a novel convenient approach for Ad6 genome modification, which is solely based on one-step in vitro recombination. This method does not involve the primary genome vectorization, the use of *E. coli* strains with active recombinase, or the preparation of a complete genome library, thus avoiding handling both unstable whole-genome plasmids and multiple plasmid/cosmid recombinations. We demonstrated that performed genetic modifications to insert the hTERT promoter and the GM-CSF transgene do not negatively affect the oncolytic potential of novel recombinant Ad6-hT-GM vector. This work may expand the potential and clinical use of Ad6-based virotherapy to meet the pressing and continued need for treatment of cancer patients.

## Data Availability

Genomic nucleotide sequence for HAdV-C6 obtained within this study have been submitted to the GenBank database under Accession Number OP871032.

## Supplementary Materials

The following supporting information can be downloaded at: https://www.mdpi.com/article/10.3390/v15010182/s1. Table S1: Primers and probes used in the study.
