# Supplementary material for: Development of Oncolytic Vectors Based on Human Adenovirus Type 6 for Cancer Treatment
Source: Viruses. 2023 Jan 7;15(1):182. doi: 10.3390/v15010182 (PMC9865941; doi:10.3390/v15010182)
Supplement: Supplementary file 1 [file viruses-15-00182-s001.zip › viruses-2102910-supplementary.pdf]

Table S1. Primers and probes used in the study

№	Primer name	Sequence 5′ → 3′
1	Ad6-LITR-for	TTCGTCTTCAAGAATTGCGATCGCATCATCAATAATATACCTTATTTTGG
2	Ad6-LITR-rev	TCACCTCCTAACACAAACTCCTCACCCTCTTC
3	Ad6-RITR-for	TTGTGTTAGGAGGTGAGCTCAACTCC
4	Ad6-RITR-rev	TACCGCATTAAAGCTGCGATCGCATCATCAATAATATACCTTATTTTG
5	pShuttle-TERT-for	CGCGCTGCAGGCCACCATGAGACATATTATCTGCCAC
6	pShuttle-TERT-rev	AATCGGATCCGCGGCCCTAGACAA
7	TERT-pShuttle-for	GGCCGCGGATCCGATTCGACCTCTCTC
8	TERT-pShuttle-rev	TGGTGGCCTGCAGCGCGGGGGTGGCCG
9	pShuttle-GMCSF-for	GTGAAGCTATTTTCCGCTTGCTTGTATTC
10	pShuttle-GMCSF-rev	TGCAGCCACATATACTTAATTAAAAAATCCCAGC
11	GMCSF-pShuttle-for	AGTATATGTGGCTGCAGAGCCTG
12	GMCSF-pShuttle-rev	CGGAAAATAGCTTCACTCCTGGACTG
13	pShuttle-ClaI-Ad6	GTGCTGGGATTTTTTAATTAAGTATATGTGG
14	pShuttle-PacI-Ad6	GTGGCAGGTAAGATCGATCACCT
15	E3for	CAGTTTATTCCCAACTTTGACGC
16	TPL3for	CGTCTAACCAGTCACAGTCG
17	GMCSFrev	CAAGAGCAGCAGGCTCTG
18	6.7rev	CAAGCTTGTAGAGTTACTTGAATTG
19	ADPrev	GTTGTGGGCGCGATGGTT
20	Probe	(Cy5)-CTGCCGGGAACGTACGAGTGCGT -(BHQ2)
21	GAPDHfor	GACAGTCAGCCGCATCTTCT
22	GAPDHrev	TTAAAAGCAGCCCTGGTGAC
23	GAPDHprobe	(Cy5)-CGTCGCCAGCCGAGCCACATC -(BHQ2)
24	E4for	GGAGTGCGCCGAGACAAC
25	E4rev	ACTACGTCCGGCGTTCCAT
26	E4probe	(Cy5)-TGGCATGACACTACGACCAACACGATCT-(BHQ2)
